# The Use of Multimode Data Collection in Random Digit Dialing Cell Phone Surveys for Young Adults: Feasibility Study

**DOI:** 10.2196/31545

**Published:** 2021-12-20

**Authors:** Daniel Alexander Gundersen, Jonathan Wivagg, William J Young, Ting Yan, Cristine D Delnevo

**Affiliations:** 1 Survey and Qualitative Methods Core Division of Population Sciences Dana-Farber Cancer Institute Boston, MA United States; 2 Westat Rockville, MD United States; 3 Center for Tobacco Studies Rutgers, The State University of New Jersey New Brunswick, NJ United States; 4 Rutgers Cancer Institute of New Jersey New Brunswick, NJ United States

**Keywords:** web mode, web survey, random digit dialing, mixed mode surveys, survey methodology, data capture, research methods, recruitment, survey, feasibility, smoking

## Abstract

**Background:**

Young adults’ early adoption of new cell phone technologies have created challenges to survey recruitment but offer opportunities to combine random digit dialing (RDD) sampling with web mode data collection. The National Young Adult Health Survey was designed to test the feasibility of this methodology.

**Objective:**

In this study, we compared response rates across the telephone mode and web mode, assessed sample representativeness, examined design effects (DEFFs), and compared cigarette smoking prevalence to a gold standard national survey.

**Methods:**

We conducted a survey experiment where the sampling frame was randomized to single-mode telephone interviews, telephone-to-web sequential mixed mode, and single-mode web survey. A total of 831 respondents aged 18 to 34 years were recruited via RDD at baseline. A soft launch was conducted prior to main launch. We compared the web mode to the telephone modes (ie, single-mode and mixed mode) at wave 1 based on the American Association for Public Opinion Research response rate 3 for screening and extended surveys. Base-weighted demographic distributions were compared to the American Community Survey. The sample was calibrated to the US Census Bureau's American Community Survey to calculate DEFFs and to compare cigarette smoking prevalence to the National Health Interview Survey. Prevalence estimates are estimated with sampling weights and are presented with unweighted sample sizes. Consistency of estimates was judged by 95% CI.

**Results:**

The American Association for Public Opinion Research response rate 3 was higher in the telephone mode than in the web mode (24% and 30% vs 6.1% and 12.5%, for soft launch and main launch, respectively), which was reflected in response rate 3 for screening and extended surveys. During the soft launch, the extended survey and eligibility rate were low for respondents pushed to the web mode. To boost productivity and survey completes for the web condition, the main launch used cell phone numbers from the sampling frame where the sample vendor matched the number to auxiliary data, which suggested that the number likely belonged to an adult in the target age range. This increased the eligibility rate, but the screener response rate was lower. Compared to population distribution from the US Census Bureau, the telephone mode overrepresented men (57.1% [unweighted n=412] vs 50.9%) and those enrolled in college (40.3% [unweighted n=269] vs 23.8%); it also underrepresented those with a Bachelor of Arts or Science (34.4% [unweighted n=239] vs 55%). The web mode overrepresented White, non-Latinos (70.7% [unweighted n=90] vs 54.4%) and those with some college education (30.4% [unweighted n=40] vs 7.6%); it also underrepresented Latinos (13.6% [unweighted n=20] vs 20.7%) and those with a high school or General Education Development diploma (15.3% [unweighted n=20] vs 29.3%). The DEFF measure was 1.28 (subpopulation range 0.96-1.93). The National Young Adult Health Survey cigarette smoking prevalence was consistent with the National Health Interview Survey overall (15%, CI 12.4%-18% [unweighted 149/831] vs 13.5%, CI 12.3%-14.7% [unweighted 823/5552]), with notable deviation among 18- to 24-year-olds (15.6%, CI 11.3%-22.2% [unweighted 51/337] vs 8.7%, CI 7.1%-10.6% [unweighted 167/1647]), and those with education levels lower than Bachelor of Arts or Science (24%, CI 19.3%-29.4% [unweighted 123/524] vs 17.1%, CI 15.6%-18.7% [unweighted 690/3493]).

**Conclusions:**

RDD sampling for a web survey is not feasible for young adults due to its low response rate. However, combining this methodology with RDD telephone surveys may have a great potential for including media and collecting autophotographic data in population surveys.

## Introduction

For a multitude of reasons, young adults have been a difficult and resource-demanding population on whom to conduct surveillance research using traditional sampling and data collection methods, especially for longitudinal surveys. They are highly mobile, moving at over twice the rate of other adults [1], making address-based and area-based sampling, as well as recontacting for follow-up data collection, challenging and resource-demanding. Likewise, they are much more likely than the general adult population to live in nontraditional and group housing such as military barracks or college dormitories [2]. Moreover, they are early adopters of new communication technologies that replace traditional contact modes, such as wireless substitution (eg, foregoing traditional landline telephones in favor of only owning cell phones). The combination of early adoption of wireless substitution, nontraditional housing, and high mobility has created substantial challenges to conducting both repeated cross-sectional and longitudinal telephone-based surveillance research among this important population subgroup. For example, their high and rapid rate of wireless substitution from the early 2000s through today led to a decline in participation and likely increased coverage bias in traditional random digit dialing (RDD) surveillance systems such as the Behavioral Risk Factor Surveillance System [3,4]. Likewise, their higher rate of living in institutional housing leads to greater coverage errors for many surveillance studies that use address-based or area probability sampling. Lastly, their high mobility rate means they are more difficult to locate and contact for follow-up data collection in longitudinal surveillance designs that typically rely on numerous approaches to engage participants such as mailings, keeping contact information for someone who knows the respondent (in case of difficulty recontacting), and making telephone calls. This is particularly detrimental to rapid surveillance of behaviors and health conditions that are themselves highly dynamic or occur in dynamic environments, such as tobacco use, which has seen a drastic shift in the tobacco product market with the introduction and growth of emerging products such as e-cigarettes in a historically short period of time.

Paradoxically, wireless substitution proved beneficial with respect to sampling young adults for cross-sectional telephone surveillance surveys [5]. Indeed, cell phone ownership is practically universal among young adults, and research found that cell-phone–only RDD led to a representative cross-section of the young adult population [3,6]. However, despite advances in sampling and weighting methodologies to address the wireless substitution challenges, response rates have continued to drastically decline. For example, Pew Research Center reports an average response rate of 28% in 2001 compared with only 6% in 2018 [7]. Thus, considerable challenges remain in conducting behavioral surveillance research, particularly rapid surveillance, in this population. This recent drastic decline in response rates for RDD surveys may be related to the rapid transformation of cell phone technology and, in turn, how cell phones are used, particularly among young adults who are the early adopters of this technology [8,9]. Indeed, smartphones, which have seen an incredibly rapid uptake in the population, allow users to communicate via multiple modes—voice, text, email, messaging, and through social media. People are decreasingly using the device for telephone conversations and increasingly using them to connect to the internet for communication via social media, texting, and email [9,10]. Moreover, some cell phone platforms (eg, iOS, Apple Inc) now include functions that block incoming calls that are not from telephone numbers known to the recipient [11]. Thus, RDD surveys once again are faced with major challenges related to secular shifts in technology use. However, as with wireless substitution, these challenges may create a new opportunity by providing a single device through which young adults can be sampled, recruited, and administered surveys via multiple modes for both cross-sectional and longitudinal surveillance research projects that use RDD and telephone mode. Indeed, this could save tremendous resources and lay the groundwork for the collection of new types of data if smartphones can be used for administering web surveys with data quality that is on par with telephone surveys.

We designed the National Young Adult Health Survey (NYAHS), a national longitudinal RDD survey of young adults, to compare two ways of integrating web-based data collection—sequential telephone-to-web mixed mode and single-mode web survey—against traditional single-mode telephone surveys. This paper uses the baseline data collection of the NYAHS to compare the response rates across telephone and web modes; to assess the generalizability of the sample by benchmarking the demographic distributions to population distributions; to examine the impact of sampling design and sample weighting on statistical precision; and to compare estimated cigarette smoking prevalence to the prevalence from a gold standard national survey.

## Methods

### Sampling and Recruitment

**Figure 1:**
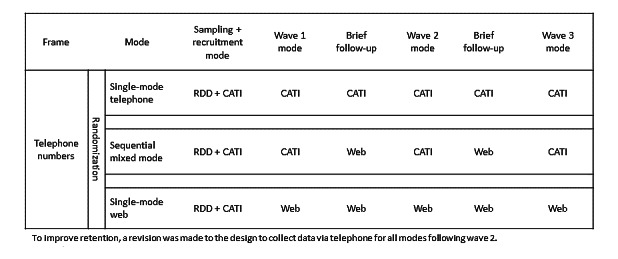
NYAHS sampling and original data collection design. CATI: computer-assisted telephone interview; NYAHS: National Young Adult Health Survey; RDD: random digit dialing.

The NYAHS was designed to provide representative cross-sectional and longitudinal estimates of health behaviors using RDD sampling and to test the feasibility and utility of integrating web surveys in surveillance systems that use RDD sampling. Specifically, we compared three modes of data collection: (1) single-mode telephone survey, (2) sequential telephone-to-web dual mode, and (3) single-mode web survey. Single-mode telephone survey is the traditional approach for RDD surveillance systems, while sequential telephone-to-web dual mode and single-mode web surveys are experimental. The sample was purchased from Marketing Systems Group, and the initial sample of 136,000 cell phone numbers was randomized to one of the three data collection modes prior to loading to the computer-assisted telephone interviewing system. Figure 1 presents the sampling and original data collection design for the study. For each mode, a random selection of cellular phone numbers from cellular-dedicated thousand-level blocks were selected. Telephone interviewers manually dialed selected numbers and read a recruitment script that explained the purpose of the study and screened for eligibility. For single-mode telephone, recruitment and each data collection occasion were conducted entirely through telephone. For the sequential mixed mode, the baseline or wave 1 survey as well as waves 2 and 3 were conducted using telephone recruitment and data collection (identical to single-mode telephone), while brief follow-up surveys were conducted via the web. For the single-mode web survey, baseline recruitment was conducted via telephone with the web mode data collection. Following baseline, invitations to web surveys were sent via text and/or email depending on the respondents’ preference.

We worked 28,519 samples as a soft launch to identify ways to streamline data collection. To ensure adequate sample size for tobacco types with lower prevalence, “ever use” of any tobacco product (ie, having used a tobacco product at any time, hereafter “ever tobacco”) was an inclusion criterion. However, in combination with lower response rates in the web condition, the ever tobacco inclusion criterion proved infeasible. Thus, for the main survey launch, the only inclusion criterion was being 18 to 34 years old. Moreover, in order to increase the likelihood that we reach eligible people in the single-mode web condition, we only used telephone numbers that were identified as having a high likelihood of belonging to young adults based on an additional preprocessing step using auxiliary data. We also made small wording changes to the recruitment script. No other changes were made to the recruitment process or data collection for either of the conditions.

### Instrumentation

The survey instrument was designed to collect data to estimate annual tobacco prevalence and longitudinal tobacco use trajectories. Consequently, most items in the survey asked about the use of tobacco and nicotine products, but the survey also asked about the awareness of new and emerging tobacco products, cannabis and alcohol use, and several demographic characteristics. We used standardized measures when possible and adapted nonstandardized measures from other tobacco surveys. The English language survey was translated into Spanish by a bilingual researcher and independently reviewed by a second translator with inconsistencies adjudicated by the investigator team. New survey questions were pretested in Spanish and English using cognitive interviewing techniques, and a usability test was carried out on iOS and Android (Google Inc) devices.

### Data Collection

Baseline data collection occurred between April 2018 and May 2019 via a computer-assisted telephone interviewing system and web-based survey. A screening questionnaire was used to identify eligible participants, defined as young adults between the ages of 18 and 34 years. The respondents in the single-mode telephone and sequential telephone-to-web modes were immediately transitioned to the main interview following the recruitment script and eligibility determination, while the respondents in the single-mode web group were texted a link to a URL for the web survey. The URL led them to a consent form page after which the respondents were taken to the main survey. Interviews and web surveys were conducted in Spanish when language barriers were encountered during recruitment. The interviews took an average of 16.8 minutes to complete and were comparable in length in both English and Spanish. Invitation texts and emails were sent in Spanish for Spanish-speaking respondents. The participants were offered a conditional US $15 electronic gift card to a major online retailer as an incentive. The data collection protocol was approved by the Institutional Review Board at Rutgers Biomedical Health Sciences.

### Sampling Weights

A base weight was calculated as the product of the inverse of the probability of selection for each sample member and the number of cellular telephones on which a respondent receives calls. The base weight was adjusted for a 24% oversampling of ever tobacco users due to the inclusion criterion of the soft launch. The base weight was then calibrated via iterative proportional fitting to population numbers from the 2018 American Community Survey (ACS) [12], a large-scale survey conducted by the Census Bureau to update population estimates between decennial censuses. Specifically, the base-weighted sample was calibrated along age (18-21 years, 24-29 years, and 30-34 years), sex (male and female), education (<BA vs ≥BA), and race or ethnicity (White, non-Latino; Black, non-Latino; Asian, non-Latino; Latino; and other). Population demographic distributions were generated from weighted analysis of the 664,617 participants aged 18 to 34 years in the ACS Public Use Microdata Sample. Calibration of the NYAHS was conducted using the ‘survey’ package in R (R Core Team) [13,14].

### Statistical Analysis

The eligibility rate was calculated as the number of respondents meeting the inclusion criteria (aged 18-34 years and ever using tobacco during the soft launch; for the main launch, just ages 18-34 years) divided by the total number of respondents who were contacted and who answered the screening questions about age (and tobacco use early in the study). Second, we calculated the American Association for Public Opinion Research response rate 3 for screening, for extended surveys, and overall [15]. The screening response rate 3 is calculated as the number of completed screeners (eligibility questions) divided by the estimated number of sampled phone numbers active and used for personal calls. Importantly, it includes in the denominator an estimate of the eligible numbers among the dialed numbers where eligibility could not be determined. Since a vast number of the dialed numbers fall in this category, it is instructive to look at the overall response rates as well as the extended survey (ie, among those for whom eligibility could be established), the latter of which is also known as cooperation rate.

Sample representativeness across data collection modes was assessed by comparing the base-weighted NYAHS demographic distribution for sex (male and female), age (18-21 years, 22-24 years, 25-29 years, and 30-34 years), and race or ethnicity (White, non-Latino; Black, non-Latino; Asian, non-Latino; Latino; and other) against population distribution from the ACS. Single-mode telephone and sequential telephone-to-web mixed mode was collapsed as their protocols are identical at baseline. Because the calibration of base-weighted demographic distributions to population values is based only on point estimates, we did not present confidence intervals as a measure of precision. Rather, we used the National Center for Health Statistics Data Presentation Standards for Proportions criteria for reliability to suppress data that are not reliable [16].

The impact of sampling design and calibration on statistical precision is summarized with the sampling design effect (DEFF). DEFF is the ratio of variance under the complex sampling design to the variance under a simple random sample for a given estimate. We calculated DEFF as presented by Kish [17], who incorporates the correlation of sampling weights with the outcome, and presented it overall and by key subpopulations.

To assess bias in cigarette smoking prevalence, we compared the estimated prevalence from the NYAHS combined across data collection modes to the National Health Interview Survey (NHIS) [18]. The NHIS is an in-person interviewer-mediated national survey that uses area probability sampling. Thus, it is not subject to any bias resulting from an RDD approach or due to push-to-web survey following telephone recruitment. Cigarette smoking was selected as a comparison benchmark as its measurement has long been standardized. By contrast, newer products do not have uniform question wording across surveillance systems; thus, they lack a gold standard measurement. Current smokers were defined as individuals who have smoked at least 100 cigarettes in their lifetime and who currently smoke every day or some days. We presented point estimates with 95% CI calculated using the logit method. The NHIS and NYAHS estimates were generated using final sampling weights and are presented with unweighted numerators and denominators. Consistency of prevalence estimates were judged by comparing the point estimates and the degree of overlap of confidence intervals. The estimates were suppressed from tables and figures if they did not meet the National Center for Health Statistics Data Presentation Standards for Proportions [16]. All analyses were conducted using the survey package in R [13,14].

## Results

Eligibility and response rates are presented in Table 1, for the main data collection (ie, after including the supplemental sampling frame for the single-mode web survey and implementing minor tweaks to the introductory language) and the soft launch. The eligibility rate for telephone was 12% (143/1191) for the soft launch and 17.6% (864/4909) for the main data collection, and 9.6% (51/532) and 30.3% (306/1009), respectively, for the single-mode web survey (where only cell phone numbers identified as having a higher likelihood of being in the 18-34 years age range were used). The screening response rate was 32.8% (4909/14980) and 39.8% (1191/2994) for the main and soft launch, respectively, compared to 16.1% (1009/6248) and 42.6% (532/1248) for the single-mode web survey. The extended response rate was 68.6% (593/864) and 74.8% (107/143) in the main and soft launch for the telephone survey and 37.9% (116/306) and 29.4% (15/51), respectively, for the single-mode web survey. These resulted in the overall response rates of 24% (main) and 30% (soft launch) for telephone and 6.1% (main) and 12.5% (soft launch) for the single-mode web survey.

**Table 1:** Response rate by mode from the National Young Adult Health Survey.

Eligibility and response rate	Soft launch			Main		
	Phone	Mixed mode	Web	Phone	Mixed mode	Web
Eligibility rate, n/N (%)	71/588 (12.1)	72/603 (11.9)	51/532 (9.6)	448/2393 (18.7)	416/2516 (16.5)	306/1009 (30.3)
Screener response rate 3, n/N (%)	588/1496 (39.3)	603/1498 (40.3)	532/1248 (42.6)	2393/7763 (30.8)	2516/7218 (34.8)	1009/6248 (16.1)
Extended response rate 3, n/N (%)	55/71 (77.5)	52/72 (72.2)	15/51 (29.4)	306/448 (68.3)	287/416 (69)	116/306 (37.9)
Response rate 3, %	30.4	29.1	12.5	21.1	24	6.1

The base-weighted sample demographics for telephone and single-mode web survey benchmarked against the 2018 ACS are presented in Table 2. The telephone survey tracked the overall population distribution well by race or ethnicity and age. The NYAHS telephone survey slightly overrepresented men (57.1% [unweighted n=412] vs 50.9%) and those currently enrolled in college (40.3% [unweighted n=269] vs 23.8%) and underrepresented those with a Bachelor of Arts or Science (34.4% [unweighted n=239] vs 55%). The single-mode web sample closely tracked the population based on gender, but overrepresented White, non-Latinos (70.7% [unweighted n=90] vs 54.4%) and those with some college education (30.4% [unweighted n=40] vs 7.6%) and underrepresented Latinos (13.6% [unweighted n=20] vs 20.7%), and those with a high school or General Education Development diploma (15.3% [unweighted n=20] vs 29.3%).

**Table 2:** National Young Adult Health Survey demographic distribution (base weighted) benchmarked to the 2018 American Community Survey for 18- to 34-year-olds.

Demographics and category		Census (ACS^a^)	Phone or mixed (unweighted N=700)	Web (unweighted N=131)
**Gender, unweighted n (weighted %)**				
	Female	49.1	288 (42.9)	58 (45.4)
	Male	50.9	412 (57.1)	73 (54.6)
**Age (years), unweighted n (weighted %)**				
	18-24	40.4	309 (45.5)	28 (22.0)
	25-29	30.6	188 (26.4)	49 (37.3)
	30-34	29.0	203 (28.1)	54 (40.7)
**Race or ethnicity, unweighted n (weighted %)**				
	Asian, non-Latino	6.5	55 (8.6)	N/A^b^
	Black, non-Latino	14.3	80 (11.8)	11 (8.0)
	Latino	20.7	148 (21.2)	20 (13.6)
	Other, non-Latino	4.1	44 (6.2)	N/A
	White, non-Latino	54.4	373 (52.2)	90 (70.7)
**Education, unweighted n (weighted %)**				
	<HS^c^	8.2	58 (8.1)	N/A
	HS or GED^d^	29.3	146 (20.9)	20 (15.3)
	Some college	7.6	257 (36.7)	40 (30.4)
	BA^e^ or BS^f^	55.0	239 (34.4)	68 (51.8)
**Currently enrolled in college, unweighted n (weighted %)**				
	Yes	23.8	269 (40.3)	39 (30.8)
	No	76.2	431 (59.7)	92 (69.2)

^a^ACS: American Community Survey.

^b^N/A: not applicable (does not meet National Center for Health Statistics Data Presentation Standards for Proportions).

^c^HS: high school.

^d^GED: General Educational Development.

^e^BA: Bachelor of Arts.

^f^BS: Bachelor of Science.

Table 3 displays the DEFF overall and by subpopulations. Overall, the estimated variance of the sampling distribution was 1.28 times greater than if the same estimate had been derived from a simple random sample. This corresponds to an approximately 13% inflation of the estimated standard errors compared with simple random sampling. DEFF ranged from a low of 0.96 among those with a Bachelor of Arts or Science to 1.93 among those with Some College, corresponding to a range of 2% decrease to 39% increase in standard errors compared to simple random samples, respectively.

**Table 3:** Design effect by subpopulations.

Demographics		DEFF^a^
**Gender**		
	Female	1.29
	Male	1.38
**Age (years)**		
	18 to 24	1.60
	25 to 29	1.25
	30 to 34	1.27
**Race or ethnicity**		
	Asian, non-Latino	1.29
	Black, non-Latino	1.50
	Latino	1.25
	Other, non-Latino	1.08
	White, non-Latino	1.28
**Education**		
	<HS^b^	1.11
	HS or GED^c^	1.10
	Some college	1.93
	BA^d^ or BS^e^	0.96
Overall		1.28

^a^DEFF: design effect.

^b^HS: high school.

^c^GED: General Education Development.

^d^BA: Bachelor of Arts.

^e^BS: Bachelor of Science.

**Figure 2:**
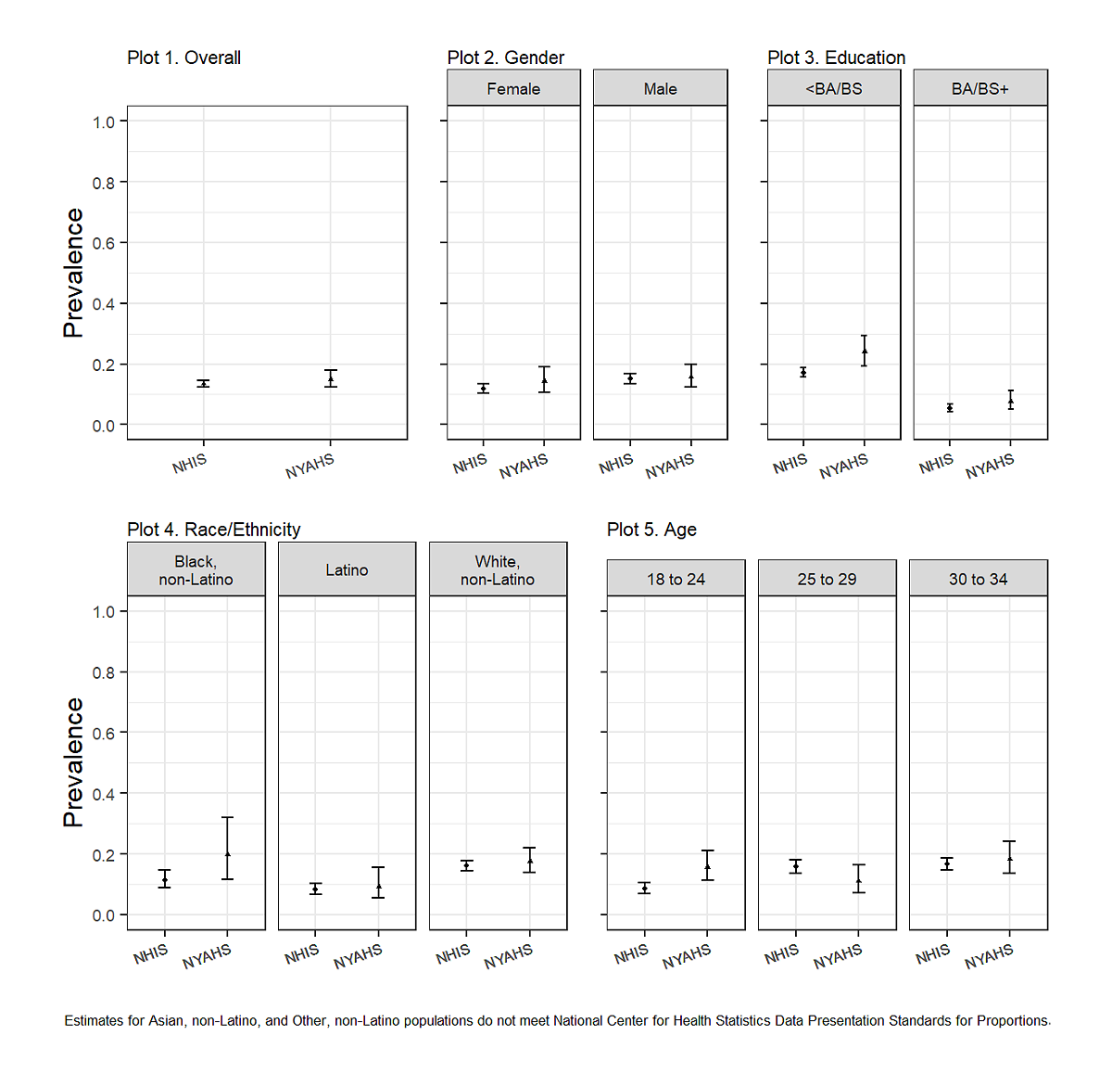
Cigarette smoking prevalence, NYAHS vs NHIS. BA: Bachelor of Arts; BS: Bachelor of Science; NHIS: National Health Interview Survey (2018); NYAHS: National Young Adult Health Survey (2018).

Figure 2 shows the estimated smoking prevalence for the NYAHS and NHIS overall (plot 1) and population subgroups (plots 2 through 5). The estimated prevalence was consistent overall (NYAHS: 15%, 95% CI 12.4%-18% [unweighted 149/831]; NHIS: 13.5%, 95% CI 12.3%-14.7% [unweighted 823/5552]), and for men, women, Whites, Latinos, Black, non-Latinos, 30- to 34-year-olds, among those with education levels <Bachelor of Arts or Science, and those with Bachelor of Arts or Science. There were notable differences only among 18- to 24-year-olds (NYAHS: 15.6%, CI 11.3%-22.2% [unweighted 51/337]; NHIS: 8.7%, CI 7.1%-10.6% [unweighted 167/1647]), and those with education levels <Bachelor of Arts or Science (NYAHS: 24%, CI 19.3%-29.4% [unweighted 123/524]; NHIS: 17.1%, CI 15.6%-18.7% [unweighted 690/3494]).

## Discussion

### Principal Findings

Our analysis demonstrated that using RDD sampling and telephone recruitment to a single-mode web survey is not feasible as a sole methodology for surveillance research, but it suggests that web surveys may have good utility using concurrent mixed mode designs (ie, concurrently conducting telephone and web surveys with different respondents) for surveillance research that use RDD sampling and telephone data collection mode. Our soft launch found that eligibility rates, including the use of tobacco as a qualifier, were so low that it proved cost prohibitive using traditional RDD sampling frames. Removing the requirement for tobacco use improved the recruitment using RDD sampling, though low response rates and the limited age eligibility range still made this a resource-intensive effort. In particular, response rates to the web survey among respondents screened as eligible were very low, though they were similar to those reported by Pew [7], which is consistent with other push-to-web research projects [19]. To increase web survey completes, the main baseline launch used an additional preprocessing step of the sampling frame to increase the likelihood of reaching a young adult. Indeed, this nearly tripled the eligibility rate.

Our methodology had good representativeness for the telephone mode and, to a lesser extent, the web mode. For the web condition, the base-weighted sampling distribution tracked the population distribution well on most characteristics, except for overrepresenting older young adults and White, non-Latinos. By contrast, the telephone sample tracked the population very well across all demographics, except college enrollment. This suggests that the preprocessed list-assisted RDD sampling frame we used for the web mode may have greater coverage error and/or differential nonresponse compared with the telephone mode. However, deviation from population distributions is common in surveys with probability sampling designs and can be addressed via weighting adjustments such as poststratification or, as in our case, calibration methods [20]. Indeed, the pooled web and telephone surveys produced unbiased cigarette estimates following calibration. Notable exceptions were for 18- to 24-year-olds and those with less than a bachelor’s degree, though this may be due to differences in the NHIS’s poststratification and our sample calibration approaches. In particular, the NHIS does not poststratify on college enrollment, which is known to be associated with tobacco use [21], while the NYAHS does [22]. Moreover, our approach had DEFFs that were considerably smaller than the Behavioral Risk Factor Surveillance System’s national estimates of 4.49 (although that estimate varies from year to year) [23]. This ensured reasonable precision for overall and some subpopulation estimates. However, larger sample sizes may be necessary for producing estimates with adequate statistical precision for smaller subgroups, particularly with distributions that deviate substantially from the population as larger weighting adjustments may result in increased variability of sampling weights and smaller effective sample sizes. Because the web mode had poorer representativeness, sample calibration and thus precision may be impacted by the proportion of the sample that is allocated to the web mode in concurrent mixed mode surveillance designs. In our case, approximately 16% (n=131) of the total sample came from the web survey, but future research should be carried out to identify the optimal allocation of the web mode and telephone mode in order to maximize the statistical precision relative to cost. The integration of a web mode in RDD methodology has tremendous potential to collect data not possible in traditional telephone surveys and/or conducting mobile-based research that is difficult to implement using probability-based sampling designs. For example, including web surveys in a telephone-based surveillance methodology allows researchers to embed visual and auditory media [24,25]. This can be used to test social marketing messaging on a large and geographically diverse scale, which can help inform public health workers and policy makers about the effective approaches to health promotion messaging. Similarly, it allows respondents to submit media in autophotography data collection to capture important contextual or exposure information that may not be adequately captured by closed or open-ended questions [26]. Such methodologies have traditionally been used in qualitative research [27,28], but future research should explore ways in which combining web surveys with RDD sampling may be used to collect such data in large-scale surveys that use probability sampling methods. Similarly, our methodology suggests that RDD sampling and the web mode may be combined with passive data collection from health tracking data linked to the respondents’ mobile devices, which may reduce the overall data collection burden on them. To date, however, the feasibility and challenges of linking survey responses to health trackers in general population surveys are not known. Future research should evaluate the receptivity of respondents to participate in pulse surveys, link the resulting information with tracking data, and identify challenges and ways to address them.

### Limitations

The limitations of this paper are that representativeness was evaluated over a relatively limited number of demographic characteristics. Ours were chosen because they are the most commonly used in poststratification or calibration and are priority groups for tobacco control. Moreover, consistency of prevalence estimates should be evaluated for other health indicators. This can be challenging because many tobacco products, particularly new and emerging tobacco products, do not have standardized measurements across surveillance systems. Thus, we would not be able to disentangle differences due to measurement versus sampling and data collection mode.

### Conclusions

Our findings demonstrated that integrating the web mode into the traditional telephone mode in a concurrent mixed mode design for surveillance research is challenging, and RDD sampling for web-based surveillance methodology may not be feasible as a sole data collection methodology. However, our findings also suggested that the web mode can be integrated in an RDD telephone surveillance system by allocating a random subsample to complete the same survey on the web. This has tremendous potential to enhance data collection, particularly for testing social marketing messages and combining general population surveys with autophotographic or health tracking data.

## Abbreviations

ACS: American Community Survey
DEFF: design effect
NHIS: National Health Interview Survey
NYAHS: National Young Adult Health Survey
RDD: random digit dialing
